# The Genus *Ochrobactrum* as Major Opportunistic Pathogens

**DOI:** 10.3390/microorganisms8111797

**Published:** 2020-11-16

**Authors:** Michael P. Ryan, J. Tony Pembroke

**Affiliations:** 1 Department of Applied Sciences, Limerick Institute of Technology, Moylish V94 EC5T, Limerick, Ireland; michaelpryan1983@gmail.com; 2 Molecular Biochemistry Laboratory, Department of Chemical Sciences, School of Natural Sciences, Bernal Institute, University of Limerick, Limerick V94 T9PX2, Ireland

**Keywords:** *Ochrobactrum*, nosocomial infection, environmental bacteria

## Abstract

*Ochrobactrum* species are non-enteric, Gram-negative organisms that are closely related to the genus *Brucella*. Since the designation of the genus in 1988, several distinct species have now been characterised and implicated as opportunistic pathogens in multiple outbreaks. Here, we examine the genus, its members, diagnostic tools used for identification, data from recent *Ochrobactrum* whole genome sequencing and the pathogenicity associated with reported *Ochrobactrum* infections. This review identified 128 instances of *Ochrobactrum* spp. infections that have been discussed in the literature. These findings indicate that infection review programs should consider investigation of possible *Ochrobactrum* spp. outbreaks if these bacteria are clinically isolated in more than one patient and that *Ochrobactrum* spp. are more important pathogens than previously thought.

## 1. Introduction

Gram-negative, non-fermenting bacteria are an emergent worry in medical situations and are becoming a growing cause of severe infections. Pathogens of this type are opportunistic and include many different bacterial species, such as *Ralstonia* spp., *Pseudomonas aeruginosa*, *Sphingomonas paucimobilis* and *Brevundimonas* spp. [1,2,3,4,5]. Gram-negative, non-fermenting bacteria can infect both patients undergoing treatments and individuals outside of a clinical setting with various underlying conditions or diseases. Another type of these bacteria are the members of the α-proteobacterial genus *Ochrobactrum* [6]. 

*Ochrobactrum* spp. are found in a wide variety of environments including water, aircraft water, soil, plants and animals [6,7,8,9,10,11,12]. Several *Ochrobactrum* spp. have been investigated for their potential to degrade xenobiotic pollutants and for heavy metal detoxification under a variety of environmental conditions [13,14,15,16]. *Ochrobactrum* spp. are very closely related to brucellae, and even though they are considered to be of low virulence, they have increasingly been found to cause infections (some serious including endocarditis and septicaemia) in immunocompetent hosts [17,18].

Investigation of the scientific/medical literature presented a wide variety of infections resultant from *Ochrobactrum* spp. and these were resistant to wide variety of antibiotics. Our data point to the genus being a more common pathogen than previously supposed, with many of the infections/conditions caused by *Ochrobactrum* spp. being aggressive and debilitating. The overall aim of this work is to present a summary of the types of *Ochrobactrum* spp. infections, any underlying disorders/illnesses in patients that accompany these infections and the potential treatments that can be used in the management of infections to support medical specialists.

## 2. Genus *Ochrobactrum*

**Figure 1:**
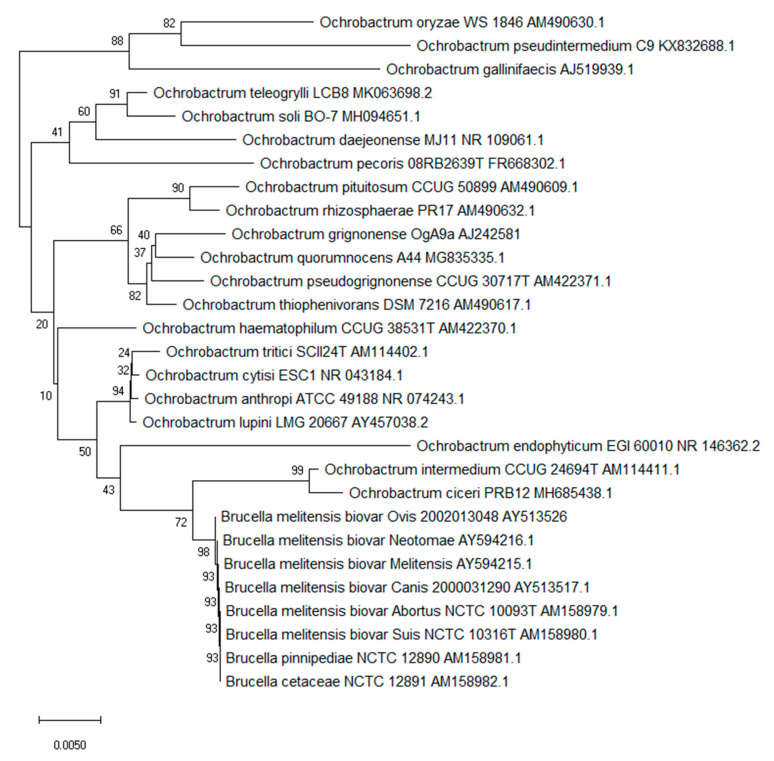
Phylogenetic structure of the genus *Ochrobactrum* along with the genus *Brucella*. The tree based on partial 16S rRNA gene sequences obtained using neighbour joining with Maximum Composite Likelihood method (MEGA package). GenBank accession numbers are given with the species name. Numbers at nodes are bootstrap values based on 1000 resamplings. Bar, 0.0050 substitutions per site [41,42].

The genus *Ochrobactrum* emerged from what was previously categorised as the CDC group VD1-2. The type species *Ochrobactrum anthropi* had previous been called *Achromobacter* VD based on the Special Bacteriology Section of the US Center for Disease Control [19]. Initial results indicate members of the group grew on MacConkey agar producing catalase, oxidase and urease; strains could be Gram-negative to variable [20]. However, the taxonomic position of *Achromobacter* became complicated and the name *Achromobacter* and related CDC group VD were no longer accepted by Bergeys Manual [19] leading to a new classification and the emergence of the genus *Ochrobactrum* [21]. *Ochrobactrum* spp. are phylogenetically related to members of the alpha-2 subdivision of *Proteobacteria*. They are catalogued on the *Brucella* rRNA branch of rRNA superfamily IV. Thus, from the previous CDC group Vd, a novel genus and a new species, *Ochrobactrum anthropi*, was proposed [21,22]. The type strain was Gram-negative, aerobic, rod shaped, non-pigmented and motile. It produced acid from a selection of carbohydrates and reduced both nitrate and nitrite and possessed a GC ratio between 56 to 59% [21]. Almost all 56 strains categorised as CDC GroupVD that were used to support the new genus *Ochrobactrum* came from various human clinical specimens. Since the initial description of *O. anthropi*, several other species have since been described (Table 1 and Figure 1). Certain *Ochrobactrum* spp. can be opportunistic pathogens especially in a hospital environment with the majority of reported cases due to hospital-acquired infections in patients with indwelling and invasive medical devices, including central venous catheters and drainage tubes [23]. In addition, the organism shows widespread resistance to penicillins and other antibiotics that cause clinical management issues with immunocompromised hosts [24,25]. The phylogenetic relationship between all described *Ochrobactrum* spp. can be seen in Figure 1.

## 3. Identification of *Ochrobactrum* spp.

*Ochrobactrum* species are Gram-negative and composed of short rods that are straight or slightly curved with one end flame shaped. They are generally motile and do not produce haemolysis on blood agar [43].

### 3.1. Biochemical Identification

Biochemical identification can be carried out using biochemical-testing kits such as the API 20NE or Vitek-2 (BioMèrieux, Las Balmas, France). When biochemical testing is carried out, it is normal to test isolates against *Brucella* agglutinating sera to prevent misdiagnosis with *Brucella* its close neighbour [44]. It has been shown that commercial kits are generally unsuitable for identification or differentiation amongst *Ochrobactrum* [45]. Analysis of 103 clinically relevant *Ochrobactrum* strains indicated that biochemical reaction profiles of the API and BD Phoenix™ 100 systems for identifying *Ochrobactrum* isolates can only be used at the genus level [46]. Care is required when identifying *Ochrobactrum* in clinical situations as misidentification has occurred with *Brucella melitensis* [47]. 

For identification of *Ochrobactrum* spp., it was proposed that the isolation of non-fastidious, non-fermenting, oxidase-positive, Gram-negative rods that are resistant to Beta-lactams (except imipenem) indicates the isolate is from the genus *Ochrobactrum* [43]. The API 20NE will confirm the identification to genus level for the majority of strains (Table 2). In addition, it has been proposed that urease activity, the mucoidy of the colonies and growth at 45 °C on tryptic soy agar coupled to susceptibility to colistin, tobramycin and netilmicin should be used as differentiating characteristics in the determination of *O. anthropi* and *O. intermedium* to the species level [43]. 

In many clinical situations, the Microscan Walkaway system is used for primary identification and any unusual non-fermentative bacteria are analysed via biochemical analysis methods such as the RapID NF Plus system. This strategy has been shown to generally perform very well [48]. There have been cases of misdiagnosis of *Ochrobactrum anthropi* (subsequently confirmed by VITEK) as *Shewanella putrefaciens* [48]. Of course, the opposite has also been reported where a *Brucella suis* bacteraemia was mistakenly identified as *Ochrobactrum anthropi* by the VITEK 2 system [49,50]. These studies underscore the difficulty encountered in identifying unusual Gram-negative, non-fermentative bacteria such as *Ochrobactrum*.

### 3.2. Fatty Acid Analysis

Use of fatty acid analysis as a differentiation marker using the Sherlock System and comparison with the Sherlock database provided the identification result for *O. anthropi* with an ID score of 0.556, indicating its poor utility for differentiation at the species level [45].

### 3.3. Molecular Identification

Molecular tools have long been applied to the typing of *Ochrobactrum* species. Early studies utilised pulsed-field gel electrophoresis and rep-PCR for the epidemiological analysis [52] followed by AFLP (Amplified Restriction Fragment Length Polymorphism) to confirm the relatedness of *O. anthropi* and *O. intermedium* with its *Brucella* relatives [53] using a limited number of isolates. The molecular diversity of a larger number of *Ochrobactrum* strains were investigated by comparing environmental isolates from soil and the rhizoplane and comparing these to a number of clinical isolates [12]. Rep-PCR using a combination of BOX and REP primers were used to profile the isolates. The isolates used in this study clustered according to their species designation [12] indicating that rep-PCR profiling offered a good tool for species delineation.

However, the differentiation of species is somewhat difficult because of their phenotypic similarity and indeed confusion amongst 16s rDNA sequences [45]. Errors still occur such as in the case of bacteraemia where the causative agent was recognised as *Ralstonia paucula* by the Microscan Walkaway system but later following DNA sequencing was identified as *O. anthropi* [54].

16s rDNA sequence similarity between *O. anthropi* and *O. intermedium* ranged from 97.9% to 98.7% depending on the strains compared [43] suggesting a higher genetic deviation in *O. intermedium* than is found in *O. anthropi*. The genetic structure of a collection of 65 isolates (37 clinical, 11 environmental and 17 from culture collections) illustrative of the known natural distribution of *O. intermedium* was analysed by MLSA (Multi-Locus Sequence Analysis) [53]. 

A *recA*-PCR RFLP (Restriction Fragment Length Polymorphism) assay was also developed to study interspecies variability within *Ochrobactrum* using *rec*A sequences from known isolates including 38 *O. anthropi* strains and type strains of *O. intermedium*, *O. tritici* and *O. lupini* and comparing these with closely related *Brucella* strains [54]. It was concluded that *recA*-sequence analysis provided a reliable molecular subtyping tool for *Ochrobactrum* at both the inter- and intraspecies level. Subsequently, a sensitive recA gene-based multi-primer single-target PCR assay has been created to differentiate *O. anthropi*, *O. intermedium* and *Brucella* that had been reported to cause diagnostic difficulty (Table 3) [55,56,57]. Teyssier et al. used 35 clinical isolates and the type strains of all known *Ochrobactrum* species (all confirmed as *Ochrobactrum* species by 16s rDNA sequencing) to examine comparative identification techniques ranging from commercial kits to biochemical and ribotyping [43]. 

### 3.4. MALDI-TOF MS

MALDI-TOF MS (Matrix-Assisted Laser Desorption/Ionisation–Time-of-Flight) was initially used to identify *Ochrobactrum intermedium* from a range of difficult to identify strains as an alternative to Vitek, API or 16s rDNA sequencing in a large validation screen with some 204 genera showing discordant results from different identification methods [58]. The method has since found utility for evaluation within the *Ochrobactrum* genus. The utility of automated rep-PCR (DiversiLab^TM^ system, BioMèrieux, Las Balmas, France) and MALDI-TOF MS analysis was compared for typing of 23 *O. anthropi* clinical isolates (bacteraemias) [44]. MALDI-TOF MS evaluation clustered the 23 strains of *O. anthropi* into a single group containing four distinct subgroups at close distance, indicating a high similarity between the isolates but also its accuracy in identification [44]. The technique of MALDI-TOF MS is gaining widespread usage in clinical situations and is increasingly utilised for *Ochrobactrum* identification in the clinic [59].

## 4. *Ochrobactrum* spp. Virulence

*Ochrobactrum* spp. are considered to be of low virulence. A study carried out by Yagel et al. into the virulome of *Ochrobactrum* spp. looked at the genomes of 130 isolates [60]. These isolates were taken from clinical, environmental, animal and plant settings. The study identified a limited number of virulence factors in the majority of these isolates. They found lipid A biosynthesis genes in all genomes analysed. They also found other virulence-associated genes in the majority of isolates such as genes associated with fatty acid biosynthesis (*fabZ*), carbohydrate metabolism (*pgm* and *cgs*), cell wall biosynthesis (*wbpL*) and biofilm formation (*ricA*, 95%). Genes for other more widespread Gram-negative virulence-associated proteins were not found in these genomes [60]. 

## 5. *Ochrobactrum* spp. Outbreaks

### 5.1. Outbreak Identification

All obtainable publications (journal articles, case reports and conference proceedings) discussing *Ochrobactrum* spp. infections were recovered using the PubMed, Web of Knowledge and Google Scholar search databases from 1980 to April 2020. The terms “CDC group VD1-2”, “Ochrobactrum”, “*Ochrobactrum* spp.”, “*Ochrobactrum anthropi*” and “*Ochrobactrum intermedium*” as well as all species names listed in Table 1 were searched. Any publications that discussed infection were set aside. These papers/abstracts were then read and the required information extracted from them. This information included year, geographic location, patient information (age, sex and any underlying medical conditions), antimicrobial testing, treatment and patient outcomes where available. The references cited from these publications were also checked for any publications/reports that may not have been found during the database searches.

### 5.2. Outbreak Analysis

The results of the investigations of the literature can be seen in Table 4 and Table 5. The tables summarise year, geographic location, patient information (age, sex and any underlying medical conditions), antimicrobial testing, treatment and patient outcome. One hundred seventeen separate instances of *Ochrobactrum anthropi* infection (277 individual cases) were identified along with a further eleven instances (twelve cases) of *Ochrobactrum intermedium*, *Ochrobactrum oryzae*, *Ochrobactrum pseudogrignonense*, *Ochrobactrum pseudintermedium* and *Ochrobactrum tritici* infection. The major breakdown of *O. anthropi* related conditions were as follows: forty-six instances of bacteraemia (42%) from which three were described as “bloodstream infections” that were usually associated with catheters, fourteen instances of septicaemia/sepsis/septic shock (12%) and two further instances of biliary sepsis (2%), nine instances of endophthalmitis, eight instances of peritonitis, four instances of pneumonia (8%) and two instances each endocarditis (2%). Other infections included two cases of keratitis (2%), four of various types of abscess (neck, pelvic, pancreatic and retropharyngeal) (3%) and one instance each of “hand infection” and brain empyema (1%). There have also been multiple reported instances of *Ochrobactrum* spp. infection that have caused two or more conditions. These include bacteraemia and necrotising fasciitis, bacteraemia and pneumonia, septicaemia and peritonitis and two instances of septic shock and endocarditits. Ten cases of death associated to *Ochrobactrum* spp. infection (all *O. anthropi*) have also been reported in the literature, four with sepsis/septicaemia (one with endocarditis), two with peritonitis and one each with a bloodstream infection, pyrogenic infection, endocarditis and infection of transjugular intrahepatic portosystemic shunt. 

## 6. Factors Linked with *Ochrobactrum* spp. Infection

### 6.1. Underlying Conditions/Illness

The bulk of *Ochrobactrum* related infections (Table 4 and Table 5) had an associated underlying disorder or disease that increased patient susceptibility to infection. Multiple patients, who were afflicted with a variety of different cancers or those with kidney failure (caused by diabetes mellitus), contracted *Ochrobactrum*-related bacteraemia/septicaemia due to a catheter/undergoing dialysis. These demonstrate how *Ochrobactrum* acts as an opportunistic pathogen in immunocompromised individuals. Infections were both hospital and community acquired. This is of interest as opportunistic pathogens such as *Ochrobactrum* spp. are mostly contracted in clinical environments. It was also interesting that a high level of instances of infection, 23 separate instances, occurred where patients had no underlying health conditions.

### 6.2. Pseudo-Outbreaks

To date, six pseudo-outbreaks have been described with *Ochrobactrum* spp. (Table 4 and Table 5). These may be challenging as they may lead to unessential/unneeded treatments such as needless courses of antibiotics or patient interventions (e.g., the removal of indwelling devices including various catheter types) and can waste both time and resources in both the clinical laboratory and treatment ward settings. Pseudo-outbreaks have many possible causes including contaminated water or materials used in the clinical testing laboratory or contaminated medical solutions such as saline. Montaña et al. described how *O. anthropi* was the reason for a pseudo-outbreak in a general treatment ward in an Argentinean hospital due to contaminated collection tubes [61]. No symptoms connected with bacterial infection were observed in any patients, even though *O. anthropi* was identified in microbiological testing. The recovered bacteria were carbapenem-resistant.

## 7. Treatment of *Ochrobactrum* spp. Infections

Treatment of *Ochrobactrum* spp. infections is often problematic, due to their resistance to different families of antibiotics such as β-lactams (penicillins, cephlasporins and emerging cases of carbapenem resistance). The antibiotic susceptibility profiles of some 103 typed strains of *Ochrobactrum* were analysed using the E-test™ for 19 clinically relevant antimicrobials [46]. In general, strains were highly resistant to β-lactam antibiotics, susceptible to ciprofloxacin, and 97.1% of the strains tested were susceptible to trimethoprim/sulfamethoxazole. This suggests that ciprofloxacin and/or trimethoprim/sulfamethoxazole in combination may be useful for empirical treatment of *Ochrobactrum* infections [46]. In the majority of outbreaks described in Table 4, aminoglycoside, fluoroquinolone, carbapenem or trimethoprim/sulfamethoxazole antibiotics were used in patient treatment. In the majority of cases, these treatments were successful in curing infections. However, as can be seen in Table 4, resistance was observed in various different outbreaks to all these antibiotics. An example of this is reported in a case of *O. anthropi* bacteraemia in a patient in Japan in 2013 where susceptibility testing showed the organism to be resistant to aztreonam, ceftazidime, cefepime, ciprofloxacin, gentamicin, levofloxacin, piperacillin, piperacillin–tazobactam and trimethoprim–sulfamethoxazole [51]. There have been no controlled trials of antibiotic therapies for *Ochrobactrum* spp. infections in humans therefore treatment should be based upon the results of in vitro susceptibility testing on the isolated clinical strains. Resistance to β-lactam antibiotics (cephalosporins, cephamycins and β-lactamase inhibitors) is due to a chromosomal gene (*bla*_och_) that is similar to the Ambler class C β-lactamase gene. This gene encodes an AmpC-like enzyme that is called OCH [168]. In addition, a plasmid-borne *bla*_oxa-181_ gene has been found in some *Ochrobactrum intermedium* strains giving resistance to carbapenems [169]. Three *Ochrobactrum* spp. strains isolated from birds in Pakistan harboured aminoglycoside (*aadB*, *aadA2*, *aac6-Ib* and *strA*, *strB*) β-lactam (*bla*_och2_ and *carb2*), tetracycline (*tetG*), chloramphenicol (*floR*), sulphonamide (*sulI*) and trimethoprim (*dfrA10*) resistance genes [170]. 

## 8. Conclusions

*Ochrobactrum* spp. are not presently thought of as major pathogens. Nevertheless, as a result of our literature search, it can be seen that there have been 128 separate outbreaks of *Ochrobactrum* spp. infections reported. Thus, the consideration that they may be innocuous should in our opinion be reconsidered based on these findings. Although the genus is considered of low virulence and of lower risk compared to other non-fermenting Gram-negative bacteria such as *Pseudomonas aeruginosa*, we feel it must not be ignored as a potential cause of infections (nosocomial or otherwise) and should be included in routine screening programs in hospitals.

## Figures and Tables

**Table 1:** List of current accepted *Ochrobactrum* species.

Species	Isolation	Genome Sequences	Reference
*Ochrobactrum anthropi*	Clinical isolate	Strain: OAB; Size: 4.9 Mbp; Ref Genome: GCA_000742955.1 (41 genomes)	[22]
*Ochrobactrum ciceri*	Nodules of Cicer	No Genome	[26]
*Ochrobactrum cytisi*	Cystisus nodules	Strain: IPA7.2; Size: 5.96 Mpb; Ref Genome: GCA_001876955.1 (1 genome)	[27]
*Ochrobactrum daejeonense*	Sludge	Strain: JCM 16234; Size: 4.8 Mbp, Ref Genome: GCA_012103095.1 (1 genome)	[28]
*Ochrobactrum endophyticum*	Roots of *Glycyrrhiza*	No Genome	[29]
*Ochrobactrum gallinifaecis*	Chicken faeces	Strain: ISO196; Ref Genome: GCF_006476605.1; Size: 3.74 Mbp (1 genome)	[11]
*Ochrobactrum grignonense*	Wheat Roots	Strain: OgA9a; Size: 4.84 Mbp; Ref Genome: NZ_NNRL00000000.1 (1 genome)	[9]
*Ochrobactrum haematophilum*	Clinical Isolate	Strain: LISuc1; Size: 4.91 Mbp; Ref Genome: GCA_003550135.1 (3 genomes)	[30]
*Ochrobactrum intermedium*	Human blood	Strain: NCTC12171; Size: 4.73 Mbp; Ref Genome: GCA_900454225.1 (18 genomes)	[22]
*Ochrobactrum lupini*	Lupinus albus rhizosphere	Strain: LUP21; Size: 5.5 Mbp; Ref Genome: GCA_002252535.1 (2 genomes)	[31,32] *
*Ochrobactrum oryzae*	Rice rhizosphere	Strain: OA447; Size: 4.47 Mbp; Ref Genome: NZ_PTRC00000000.1 (1 genome)	[33]
*Ochrobactrum pecoris*	Farm Animals	Strain: 08RB2639; Size: 5.06 Mbp; Ref Genome: GCA_006376675.1 (1 genome)	[34]
*Ochrobactrum pituitosum*	Industrial Environment	Strain: AA2 Size: 5.47 Mbp; Ref Genome: GCA_002025625.1 (4 genomes)	[35]
*Ochrobactrum pseudintermedium*	Clinical isolate	Strain: CCUG34735; Size: 4.39 Mbp; Ref Genome: GCA_008932435.1 (1 genome)	[36]
*Ochrobactrum pseudogrignonense*	Clinical isolate	Strain: K8; Size: 4.99 Mbp; Ref Genome: GCA_001652485.1 (6 genomes)	[30]
*Ochrobactrum quorumnocens*	Potato rhizosphere	Strain: A44; Size: 5.5 Mbp; Ref Genome: GCA_002278035.1 (2 genomes)	[37]
*Ochrobactrum rhizosphaerae*	Potato rhizosphere	Strain: PR17; Size: 4.9 Mbp; Ref Genome: GCF_002252475.1 (2 genomes)	[38]
*Ochrobactrum soli*	Cattle farm soil	Strain: BO-7; Size: 45 Mbp; Ref Genome: GCA_003664555.1 (3 genomes)	[39]
*Ochrobactrum thiophenivorans*	Industrial Environment	Strain: DSM 7216; Size: 4.4 Mbp; Ref Genome: GCA_002252445.1 (2 genomes)	[38]
*Ochrobactrum teleogrylli*	insect *Teleogryllus occipitalis*	No Genome	[40]
*Ochrobactrum tritici*	wheat rhizosphere root soil	Strain: DSM 13340; Size: 5.5 Mbp; Ref Genome: GCA_012395245.1 (6 genomes)	[9]

* First described as *Ochrobactrum lupini* by Trujillo et al. [31] and later reclassified as *Ochrobactrum anthropi* by Volpiano et al. [32] following whole-genome sequence analysis.

**Table 2:** Phenotypic characteristics observed for *Ochrobactrum* strains based on reactivity within the API 20E Biolog GN and API 20NE systems [9,43,51].

Using the API 20E, API 20NE, Biolog GN 1	Reaction
Indole	−ve
Catalase	+ve
Cytochrome oxidase	+ve
H_2_S	-
Acetoin	−ve
Citrate utilisation	−ve
Carbohydrate fermentation	−ve
Adipate assimilation	−ve
Detection Arginine dihydrolase	−ve
Detection Lysine decarbooxylase	−ve
Detection Ornithine decarboxylase	−ve
Detection Beta galactosidase	−ve
Detection Gelatinase	−ve
Urease *O. anthropi*	+ve
*O. intermedium*	variable
*O. grignonens*	−ve
*O. tritici*	−ve
*O. gallinifaecis*	−ve
Assimilation glucose, arabinose, mannose, N-acetylglucosamine, maltose and malate	+ve

**Table 3:** Molecular methods applied to identify *Ochrobactrum* spp. [57].

Method	Target	Sequence	Amplicon (bp)	Species
PCR	*recA*	Anth-f GCAAGCTGGGTGTCGATCTGG Anth- r TTCTCGACGACACCGGCCTTTA	544	*Ochrobactrum anthropi*
PCR	*recA*	Inter-f CGGCGTTGGTGGCTTGCCTAA Inter-r GGAACGAGAGATAGACGCGGTA	402	*Ochrobactrum intermedium*

**Table 4:** Incidences of *Ochrobactrum anthropi* infection from 1980 to 2020. Main characteristics of the case reports.

Author (Ref)	Year	Sex/Age	Country	Co-Morbidity	Type of Infection	Susceptible to *	Resistance to *	Treatment	Outcome
Appelbaum and Campbell [62]	1980	M/75 years old	USA	COPD, MI, CVA	Pancreatic abscess	Gentamicin, TMP-SMZ	Amikacin, Chloramphenicol, Tetracycline, Tobramycin	Gentamicin	Died of respiratory failure
Kish [63]	1984	F/21 years old	USA	Astrocytoma	Bacteraemia (catheter related)	Amikacin, Gentamicin Imipenem, Moxalactam, Gentamicin, Tetracycline, TMP-SMZ	Chloramphenicol, Netilmicin, Rifampin, Tobramycin	TMP-SMZ, Gentamicin	Complete Recovery
Barson et al. [20]	1987	M/14 years old	USA	Puncture wound of the foot	Osteochondritis	Amikacin, Gentamicin Imipenem, Moxalactam, Gentamicin, Rifampin Tetracycline, TMP-SMZ	Chloramphenicol, Netilmicin, Tobramycin	TMP-SMZ, Gentamicin	Complete Recovery
Van Horn [64]	1989	F/23 years old	USA	Hodgkin’s disease, had undergone bone marrow transplantation	Bacteraemia (catheter related), Urinary Tract Infection	Amikacin, Norfloxacin, Tetracycline, TMP-SMZ	Ampicillin, Aztreonam, Carbenicillin, Cefoperazone, Cefoxitin, Ceftazidime, Cephalothin, Chloramphenicol, Gentamicin, Mezlocillin, Piperacillin, Ticarcillin, Tobramycin	Norfloxacin (400 mg orally twice a day), TMP-SMZ (320/1600 mg orally every 6 h), Amikacin (500 mg intravenously every 12 h)	Complete Recovery
Cieslak et al. [65]	1992	F/3 years old	USA	Retinoblastoma	Bacteraemia (catheter related)	Amikacin, Ciprofloxacin, Gentamicin, Imipenem, Polymyxin E, TMP-SMZ,	Ampicillin, Aztreonam, Cefoxitin, Ceftazidime, Ceftriaxone, Cephalothin, Mezlocillin, Rifampin, Tetracycline, Tobramycin	Vancomycin and Ceftazidime Followed by Amikacin and TMP-SMZ,	Complete Recovery
Gransden et al. [66]	1992	Multiple (7 cases)	UK	Multiple	Sepsis (catheter related)	Amikacin, Ciprofloxacin, Gentamicin, Imipenem, TMP-SMZ, Tobramycin	Amoxicillin, Amoxicillin–clavulanate, Azlocillin, Aztreonam Cefuroxime, Cefotaxime, Cefoxitin, Ceftazidime, Ticarcillin Trimethoprim	Ciprofloxacin, Gentamicin, Imipenem	5 complete Recovery, 2 deaths unrelated to infection
Brivet et al. [67]	1993	F/74 years old	France	Alcoholism	Necrotising fasciitis, bacteraemia and multiorgan failure,	Amikacin, Ceftazidime, Cefotaxime, Ciprofloxacin, Imipenem, Pefloxacin, Ciprofloxacin, TMP-SMZ	Amoxicillin, Amoxicillin–clavulanic acid, Carbenicillin, Cephalothin, Colimycin, Piperacillin	Amoxicillin–clavulanic acid and amikacin. Followed by Imipenem	Complete Recovery
Kern et al. [68]	1993	Multiple (4 Cases—F 2–77 years old)	USA	Leukaemia	Bacteraemia (catheter related)	Imipenem	Ampicillin Amoxicillin–Clavulanic acid, Chloramphenicol Mezlocillin, Piperacillin	Amikacin, Ceftazidime–Netilmicin, Piperacillin–Netilmicin Catheter removal in one case	Complete Recovery
Klein & Eppes 1993 [69]	1993	F/7 years old	USA	Leukaemia	Bacteraemia (catheter related)	Amikacin, Ceftriaxone, Ciprofloxacin, Gentamicin, Imipenem, TMP-SMZ, Tobramycin	Ampicillin, Aztreonam, Ceftazidime, Cephalothin, Mezlocillin,	Gentamicin, Imipenem Catheter Removal	Complete Recovery
Alnor et al. 1994 [70]	1994	Multiple (3 Cases)	Denmark	Crohn’s disease, Gastric ulcer	Septicaemia Peritonitis (catheter related)	Ciprofloxacin, Gentamicin, Imipenem, Tetracycline	Ampicillin, Aztreonam, Cefuroxime, Cefotaxime, Ceftazidime, Ceftiaxione, Erythromycin, Nitrofurantoin, Piperacillin, Rifampin, TMP-SMZ	N/A	Complete Recovery
Ezzedine et al. [71]	1994	Multiple (5 Cases)		Organ transplant patients	Bacteraemia in rabbit anti-thymocyte globulin (RATG) infusion vials	Amikacin, Imipenem, Ofloxacin	Ampicillin, Amoxicillin–Clavulanic, Aztreonam Cefazolin, Temocillin,	N/A	Complete Recovery
Haditsch et al. [72]	1994	F/14 years old	Austria	Leukaemia	Bacteraemia	Amikacin, Polymyxin B, Imipenem, Norfloxacin, Tetracycline	Ampicillin, Amoxicillin–Clavulanic acid, Cefazolin, Cefoxitin, Ceftriaxone, Ceftazidime, Gentamicin, Piperacillin Tobramycin, TMP-SMZ	N/A	Complete Recovery
Braun et al. [73]	1996	M/66 years old	Germany	Cataract surgery	Endophthalmitis after cataract surgery	Amikacin, Ciprofloxacin, Imipenem, Tetracycline	Cephalosporins, Penicillins, Tobramycin, TMP-SMZ	Ciprofloxacin (400 mg two times daily)	Complete Recovery
Chang et al. [74]	1996	Multiple (3 Cases)	USA	Neurosurgery Patients	Meningitis	Ciprofloxacin, Gentamicin, Imipenem–cilastatin, Tetracycline	N/A	Imipenem–Cilastatin, Gentamicin (Six weeks)	Complete Recovery
Cieslak et al. [75]	1996	F/61 years old	USA	Hypertension, hypothyroidism, Renal insufficiency, chronic congestive heart failure	Pyogenic Infection	N/A	N/A	Gentamicin, Ceftazidime, Gentamicin, Vancomycin After failure TMP-SMZ	Died
Cieslak et al. [75]	1996	M/66 years old	USA	Small cell carcinoma	Pyogenic Infection	N/A	N/A	Aztreonam, Ceftazidime, Vancomycin After failure TMP-SMZ	Complete Recovery
Cieslak et al. [75]	1996	M/29 years old	USA	None	Pyogenic Infection	N/A	N/A	Cephradine	Complete Recovery
Ramos et al. [76]	1996	F/26 years old	Spain	Cancer	Bacteraemia (catheter related)	Ciprofloxacin, Gentamicin, Imipenem, TMP-SMZ	Ampicillin, Ceazolin, Cefotaxime, Ceftazidime	Ciprofloxacin (oral for 10 days)	Complete Recovery
Ramos et al. [76]	1996	F/62 years old	Spain	Cancer	Bacteraemia (catheter related)	Ciprofloxacin, Gentamicin, Imipenem, TMP-SMZ	Ampicillin, Ceazolin, Cefotaxime, Ceftazidime	Gentamicin and Catheter removal	Complete Recovery
Berman et al. [77]	1997	F/74 years old	USA	Pneumonia	Endophthalmitis with indwelling catheters for venous access	N/A	N/A	Ciprofloxacin (Oral 500 mg twice daily for 2 weeks)	Complete Recovery
Christenson et al. [78]	1997	Multiple (3 Cases)	USA	Various	Meningitis (pericardial allograft tissue)	Ciprofloxacin, Gentamicin, Imipenem, Tetracycline	Amikacin, Ampicillin, Cefotaxime. Ceftazidime, Cefuroxime. Cephalothin. Chloramphenicol, Piperacillin, Rifampin, Ticarcillin, Ticarcillin–Clavulanic acid, TMP-SMZ	Removal of tissue allograft implants	Complete Recovery
Earhart et al. [79]	1997	F/40 years old	USA	Rheumatic heart disease	Infection of retained pacemaker leads	Ciprofloxacin, Gentamicin Imipenem, TMP-SMZ	N/A	Ciprofloxacin, Rifampin Vancomycin, TMP-SMZ for 6 weeks Followed by Ciprofloxacin TMP-SMZ for 4 1/2 months	Complete Recovery
Gill et al. [80]	1997	M/45 years old	USA	Coronary artery disease	Intravenous line infection	N/A	N/A	None Administered	Complete Recovery
Torres et al. 1998 [81]	1998	N/A	Spain	AIDS	Bacteraemia	N/A	N/A	N/A	N/A
Yu et al. [82]	1998	Multiple (15 Cases) 10 Cases CA	China	Various	Bacteraemia (3 catheter related)	Amikacin, Ceftriaxone, Cefoperazone, Gentamicin, Imipenem	Ampicillin, Aztreonam, Amoxicillin–Clavulanic acid, Ceftazidime, Cephalothin, Cefonicid, Piperacillin,	Aminoglycoside	Complete Recovery
Jelveh & Cunha 1999 [6]	1999	M/33 month old CA	USA	Osteomyelitis	Bacteraemia	Gentamicin Levofloxacin, TMP-SMZ	N/A	N/A	Complete Recovery
Hay & Lo 1999 [83]	1999	F/Neonate HA	UK	Neonate	Meningitis	Amikacin, Ciprofloxacin, Gentamicin, Meropenem, Tobramycin, TMP-SMZ	Ampicillin, Amoxicillin–clavulanic acid, Aztreonam, Cefotaxime. Ceftazidime, Cefuroxime, Piperacillin–Tazobactam	Gentamicin	Complete Recovery
Inoue et al. 1999 [84]	1999	M/64 years old	Japan	None	Endophthalmitis	Amikacin, Cefmetazole, Cefbuperazone, Gentamicin, Imipenem, Levofloxacin, Minocycline, Tobramycin	Ampicillin, Piperacillin, Cefazolin, Cefotiam, Ceftazidime Flomoxef	Imipenem (Intravenously), Ciprofloxacin, Minocycline	Complete Recovery
Manfredi et al. 1999 [85]	1999	M/41 years old	Italy	HIV	Septicaemia	Amikacin, Carbapenems, Ciprofloxacin, Gentamicin, Ticarcilllin–Clavulanate TMP-SMZ	Aztreonam, Ceftazidime, Tobramycin	Meropenem (3 g/day)	Complete Recovery
Manfredi et al. [85]	1999	M/35 years old	Italy	HIV	Septicaemia	Amikacin, Carbapenems, Ceftazidime, Ciprofloxacin, Gentamicin, TMP-SMZ	Aztreonam, Ticarcilllin–Clavulanate, Tobramycin	Amikacin (500 mg/day), Ceftazidime (3 g/day)	Complete Recovery
Mastroianni et al. [86]	1999	M/47 years old	Italy	None	Bacteraemia	Amikacin, Amoxicillin Carbenicillin, Chloramphenicol, Ciprofloxacin, Gentamicin, TMP-SMZ, Tobramycin	Cephalosporins, Imipenem, Tetracycline	Ciprofloxacin (14-day course, intravenous, 400 mg/day)	Complete Recovery
Saavedra et al. [87]	1999	M/4 years old	Spain	Neuroblastoma	Bacteraemia (catheter related)	N/A	Amikacin, TMP-SMZ	Imipenem (14 days)	Complete Recovery
Stiakaki et al. [88]	1999	Multiple (9 Cases—All children)	Greece	Cancer	Bacteraemia (7 catheter related)	Amikacin, Ciprofloxacin Imipenem, TMP-SMZ	β-lactam antibiotics	Imipenem, Ciprofloxacin or catheter removal (2 cases)	Complete Recovery
Chertow 2000 [89]	2000	F/74 years old	USA	Renal Failure	Bacteraemia (catheter related-Haemodialysis)	Aminoglycosides Imipenem, Quinolones	Penicillins, Cephalosporins, TMP-SMZ	Ciprofloxacin (500 mg daily) and Tobramycin (40 mg intravenously)	Complete Recovery
Deliere et al. [90]	2000	Multiple (2 Cases) HA	France	Leukaemia	Sepsis (catheter related)	Ciprofloxacin, Colistin Imipenem, Rifampicin	Beta-lactams, Chloramphenicol, Fosfomycin	Imipenem (and catheter removal in one patient	Complete Recovery
Esteban et al. [91]	2000	F/79 years old	Spain	Diabetic nephropathy	Peritonitis in CAPD patient	Amikacin, Ciprofloxacin Gentamicin, Imipenem, Meropenem, TMP-SMZ	Ampicillin, Amoxicillin–Clavulanate, Cefazolin, Cefuroxime, Ceftriaxone, Ceftazidime, Cefepime, Piperacillin–Tazobactam, Ticarcillin	Ofloxacin (200 mg/day for 14 days)	Died (Unrelated to infection)
Mahmood et al. [92]	2000	F/39 years old	Pakistan	Asthmatic and non-insulin dependent diabetic	Infective Endocarditis and Septic Embolization	Ciprofloxacin, Gentamicin, Imipenem, Meropenem, Ofloxacin, TMP-SMZ	Ampicillin, Cefotaxime Piperacillin–Tazobactam,	Gentamicin (1 mg/kg 8-hourly)	Complete Recovery
Peltroche-Llacsahuanga et al. [93]	2000	F/39 years old	Germany	End-stage renal disease	Peritonitis in CAPD patient	Amikacin, Ciprofloxacin, Colistin Gentamicin, Imipenem, Meropenem, TMP-SMZ, Tobramycin	Ampicillin, Cefotiam, Cefotaxime, Ceftazidime, Cefepime, Mezlocillin, Piperacillin, Piperacillin–Tazobactam, Vancomycin	Imipenem (200 mg/1500 mL bag; four bags/day) and Ceftazidime (250 mg/1500 mL bag; four bags/day)	Complete Recovery
Shelly and Mortensen [94]	2000	M/2.5 years old	USA	None	Infection	Ampicillin, Gentamicin	Cefuroxime, Ceftriaxone, TMP-SMZ	Cefazolin Followed by Ampicillin–Subactam	Complete Recovery
El-Zimaity et al. [95]	2001	N/A	UK	None	Pseudo-bacteraemia	N/A	N/A	N/A	N/A
Greven et al. [96]	2001	N/A	USA	N/A	Chronic postoperative endophthalmitis	N/A	N/A	N/A	N/A
Daxboeck et al. [97]	2002	Multiple (2 Cases)	Austria	Chronic renal failure resulting from diabetic nephropathy	Bacteraemia (haemodialysis patients)	Amikacin, Ciprofloxacin Doxycycline, Gentamicin, Imipenem	β-lactam antibiotics, TMP-SMZ	Gentamicin	One patient recovered, one died due to MI
Galanakis et al. [98]	2002	Multiple (11 Cases—All less than 7 years old)	Greece	None	Bacteraemia	Amikacin, Ciprofloxacin Gentamicin, Imipenem, Nalidixic acid, Ofloxacin, Perfloxacin, Netilmicin, Norofloxacin Streptomycin, TMP-SMZ, Tobramycin	Ampicillin, Amoxicillin, Amoxicillin–Clavulanic acid, Aztreonam, Cefalothin, Cefepime, Cefotaxime, Cefuroxime, Piperacillin, Piperacillin–Tazobactam, Ticarcillin, Ticarcillin–Clavulanate	TMP-SMZ (oral delivery in one patient)	Complete Recovery
Stiakaki et al. [99]	2002	Multiple (11 Cases)	Greece	Various Cancers	Bacteraemia (catheter related)	Aminoglycosides, Colistin, Imipenem, Quinolones, TMP-SMZ	Ampicillin, Amoxicillin, Amoxicillin–Clavulanate, Aztreonam, Cefalothin, Cefepime, Cefotaxime Cefuroxime, Ticarcillin, Ticarcillin–Clavulanate, Piperacillin, Piperacillin–Tazobactam, Ticarcillin, Ticarcillin–Clavulanate	Various different treatments in all 11 cases	N/A
Wheen et al. 2002 [100]	2002	F/62 years old	New Zealand	None	Osteomyelitis (vertebral)	Aminoglycosides, Amoxicillin, Cephalosporins, Fluoroquinolones, TMP-SMZ	N/A	Ceftriaxone (Intravenously for 6 weeks) followed by Ciprofloxacin (orally for 6 weeks)	Complete Recovery
Gascón et al. [101]	2003	M/3 years old CA	Spain	None	Bacteraemia	Aminoglycosides, Ciprofloxacin, Imipenem, TMP-SMZ	Aztreonam, Ceftazidime Cefsulodin, Phosphomycin, Piperacillin–Tazobactam, Ticarcillin–Clavulanic acid	Gentamicin TMP-SMZ	Complete Recovery
Hill [102]	2003	N/A	UK	N/A	Pseudo-bacteraemia	N/A	N/A	N/A	N/A
Kettaneh et al. [17]	2003	F/30 years old	France	None	Septic Shock	Amikacin, Gentamicin, Imipenem, Netilmicin, Pefloxacin, Tobramycin, TMP-SMZ	N/A	Gentamicin infusion (infusion of 240 mg once), Ofloxacin (200 mg infusion twice a day for 11 days)	Complete Recovery
Oliver [48]	2003	30 years old	USA	N/A	Infection	N/A	N/A	N/A	N/A
Romero Gomez et al. [103]	2004	F/65 years old	Spain	Hypertension and rheumatic heart disease	Prosthetic mitral valve endocarditis	Aminoglycosides, Meropenem, Quinolones	β-lactams, TMP-SMZ	Meropenem (Intravenously 1 g every 6 h) and Gentamicin.	Complete Recovery
Oliver et al. [104]	2005	M/30 years old	USA	None	Bacteraemia (gunshot wound)	Amikacin, Ciprofloxacin Gentamicin, Imipenem, TMP-SMZ, Tobramycin	Aztreonam, Cefepime, Cefotaxime, Ceftazidime, Ceftiaxione, Piperacillin, Piperacillin–Tazobactam	Cefepime (2 g IV BID for 3 days) Ciprofloxacin (400 mg IV BID for 8 days) Imipenem (1 g IV for 7 days)	Complete Recovery
Cho et al. [105]	2006	F/69 years old	Korea	Hypertension	Bacteraemia (associated with medicinal plants)	Colistin, Imipenem, Meropenem, Tetracycline	Amikacin, Aztreonam, Cefepime, Ceftazidime, Cefpirome, Ciprofloxacin, Gentamicin, Isepamcin, Netilmicin, Pefloxacin, Piperacillin, Piperacillin–Tazobactam, Ticarcillin, Ticarcillin–Clavulanate, TMP-SMZ, Tobramycin	Imipenem	Complete Recovery
Ozdemir et al. [18]	2006	F/42 years old CA	Turkey	None	Endocarditis and septic shock	Amikacin, Ciprofloxacin Gentamicin, Imipenem, TMP-SMZ	β-lactams (Excluding Carbapenases) Erythromycin, Chloramphenicol	Meropenem (500 mg Twice daily) Vancomycin (500 mg Twice daily)	Died
Vaidya et al. [106]	2006	M/49 years old	USA	None	Pelvic Abscess	Gentamicin, Imipenem, Levofloxacin, TMP-SMZ	Cefepime, Tobramycin	Levofloxacin Metronidazole	Complete Recovery
Aly et al. [107]	2007	F/2 years old		Long-chain 3-hydroxyacyl-coenzyme A dehydrogenase deficiency	Bacteraemia	Ciprofloxacin, Levofloxacin, Piperacillin–Tazobactam TMP-SMZ	Amikacin, Cefotaxime, Ceftazidime, Ceftriaxone, Cefepime, Gentamicin, Imipenem Piperacillin, Tetracycline, Ticarcillin–Clavulanate, Tobramycin	Cefotaxime	Complete Recovery
Labarca et al. [108]	2007	Multiple (8 Cases)	Chile	N/A	Pseudo-bacteraemia	N/A	N/A	N/A	N/A
Lee et al. [109]	2007	M/80 years old	Korea	intrahepatic duct carcinoma	Bacteraemia	N/A	N/A	N/A	Complete Recovery
Song et al. [110]	2007	Multiple (9 Cases)	Korea	N/A	Chronic pseudophakic endophthalmitis	Ciprofloxacin, Imipenem, Ofloxacin, TMP-SMZ,	Ampicillin, Amoxicillin, Ceftazidime, Gentamicin Piperacillin, Ticarcillin, Tobramycin	N/A	Complete Recovery
Yu et al. [111]	2007	M/62 years old CA	Korea	Liver cirrhosis	Peritonitis	Amikacin, Ciprofloxacin Gentamicin, Imipenem, Levofloxacin, Meropenem, TMP-SMZ, Tobramycin	β-lactams	Imipenem (250 mg every 6 h)	Died
Arora et al. [112]	2008	M/64 years old	India	Hypertension, Diabetes, Coronary artery disease	Septicaemia (intra-aortic balloon pump (IABP) insertion)	Ciprofloxacin, Cefoperazone–Sulbactam, Imipenem, Tobramycin	Amikacin, Aztreonam, Cefotaxime, Cefoperazone, Gentamicin, Piperacillin, Ticarcillin	Ciprofloxacin (100 mL intravenous, twice a day), Cefazolin (1 g IV thrice a day), meropenem (1 g IV tds) metronidazole (100 mL IV tds)	Died
Battaglia et al. [113]	2008	M/17 years old	USA	None	Septic arthritis	N/A	N/A	Ciprofloxacin (oral 500 mg twice daily for 4 week), TMP-SMZ (Oral 160 mg/800 mg twice daily for 2 weeks)	Complete Recovery
Javaid et al. [114]	2008	M/84 years old	UK	Acute renal failure	Bacteraemia (catheter related)	Ciprofloxacin, Meropenem	Ceftazidime, Gentamicin, Ticarcillin–Clavulanate	Ciprofloxacin (250 mg orally twice a day for 2 weeks), After failure Catheter removal Meropenem (250 mg/day)	Complete Recovery
Menuet et al. [115]	2008	F/17 years old	France	Cystic Fibrosis. Diabetes	Pneumonia	Amikacin, Ciprofloxacin, Gentamicin, Imipenem, Isepamicin, Rifampicin, TMP-SMZ, Tobramycin	Amoxicillin, Amoxicillin–Clavulanate, Ceftazidime, Ceftriaxone, Colistin, Ticarcillin, Ticarcillin–Clavulanate, Piperacillin–Tazobactam	Imipenem (4 g/day) Tobramycin (2 g/day)	Complete Recovery
Chiang et al. [116]	2009	M/75 years old	Taiwan	MI	Endophthalmitis (Cataract surgery)	N/A	N/A	Ciprofloxacin	Complete Recovery
Duran et al. [117]	2009	M/Neonate	Turkey	Neonate (meconium peritonitis)	Bacteraemia (catheter related)	Ciprofloxacin, Gentamicin, Imipenem	Amikacin, Ampicillin–Sulbactam, Aztreonam, Cefepime, Ceftriaxone, TMP-SMZ Tobramycin	Ciprofloxacin Gentamicin	Died (Unrelated to *Ochrobactrum* infection)
Kim et al. [118]	2009	F/46 years old	Korea	Ovarian cancer	Bacteraemia (catheter related)	Amikacin, Colistin, Ciprofloxacin, Gentamicin, Netilmicin, Pefloxacin, TMP-SMZ, Tobramycin	Aztreonam, Ceftazidime, Cefpirome, Cefepime Meropenem, Piperacillin, Piperacillin–Tazobactam, Ticarcillin Ticarcillin–Clavulanate	Ciprofloxacin, Imipenem for 3 weeks	Complete Recovery
Ospina et al. [119]	2009	F/49 years old	Colombia	Alcoholism	Bacteraemia	Carbapenem	Ampicillin–Sulbactam	Meropenem	Complete Recovery
Rihova et al. [120]	2009	M/51 years old	Belgium	Chronic kidney disease	Peritonitis (CAPD patient)	N/A	N/A	Amikacin Meropenem	Complete Recovery
Soloaga et al. [25]	2009	M/69 years old	Argentina	Type 2 diabetes	Bacteraemia (catheter related)	Ciprofloxacin, Imipenem, Meropenem, TMP-SMZ	Amikacin, Ceftazidime, Cefepime, Gentamicin, Piperacillin–Tazobactam	Ciprofloxacin (200 mg/12 h) Meropenem (500 mg/24 h post dialysis)	Complete Recovery
Adeyemi et al. [121]	2010	N/A	Nigeria	HIV	Bloodstream infections	Amikacin, Ampicillin, Cefuroxime, Chloramphenicol, Gentamicin, Ofloxacin, TMP-SMZ	Ceftazidime, Cefotaxime, Nalidixic acid	N/A	N/A
Quintela et al. [122]	2010	F/50 years old	Spain	Terminal chronic renal failure	Peritonitis (peritoneal dialysis)	N/A	N/A	PD catheter removal	Complete Recovery
Saveli et al. [123]	2010	M/53 years old	USA	Gout, Alcoholism	Septic arthritis	N/A	N/A	TMP-SMZ (800 mg/160 mg 2 tablets every 12 h)	Complete Recovery
Sepe et al. [124]	2010	M/71 years old	Italy	Type 2 diabetes	Peritonitis (automated peritoneal dialysis)	N/A	N/A	Cefotaxime (1 g), Gentamicin (80 mg intraperitoneal)	Complete Recovery
Starr [125]	2010	N/A	USA	N/A	N/A	N/A	N/A	N/A	N/A
Wi & Peck [126]	2010	Multiple (12 Cases)	Korea	Cancer (11 cases) and Liver Cirrhosis (1 case)	Biliary sepsis (8 Cases), peritonitis (1 case), catheter-related infection (3 cases)	Amikacin, Ciprofloxacin, Gentamicin, Imipenem, Meropenem, TMP-SMZ	Aztreonam, Ceftazidime, Ceftriaxone, Cefotaxime, Piperacillin–Tazobactam,	Various	Complete Recovery (11 cases) Died (1 case)
Woo Nho et al. [127]	2010	M/66 years old	Korea	Diabetes mellitus	Peritonitis (peritoneal dialysis)	Amikacin, Ciprofloxacin, Colistin, Gentamicin, Minocycline, TMP-SMZ, Tobramycin	Aztreonam, Meropenem, All β-lactams	Amikacin, Ciprofloxacin, Meropenem catheter removal	Complete Recovery
Yagüe-Muñoz et al. [128]	2010	M/8 years old CA	Spain	Cystic fibrosis	Bacteraemia	Amikacin, Ciprofloxacin, Colistin, Gentamicin, Imipenem, Levofloxacin, Meropenem Netilmicin, TMP-SMZ Tobramycin	Ampicillin–Sulbactam, Aztreonam, Ceftazidime, Piperacillin, Piperacillin–Tazobactam, Ticarcillin	Tobramycin	Complete Recovery
Obando et al. [129]	2011	F/19 years old	Chile	Hypothyroidism, end-stage chronic renal failure	Bacteraemia (catheter related)	Amikacin, Gentamicin, Imipenem Levofloxacin, Meropenem	Ampicillin–Sulbactam, Aztreonam Ceftazidime, Cefepime,	Levofloxacin and Catheter removal	Complete Recovery
Shivaprakasha et al. [130]	2011	M/75 years old	India	Aortic valve replacement	Endocarditis (prosthetic aortic valve endocarditis)	Amikacin, Ciprofloxacin, Doripenem Gentamicin, Imipenem, Meropenem Netilmicin, TMP-SMZ	N/A	Ceftriaxone (1 g intravenous twice daily), Amikacin (1 g intravenous once daily) Followed by Meropenem (500 mg 8 hourly)	Died
Chan & Holland [131]	2012	F/21 years old	USA	Asthma, hypertension, gastric reflux	Endophthalmitis (Boston type 1 keratoprosthesis implantation)	N/A	N/A	Levofloxacin	Complete Recovery
Shrishrimal [132]	2012	M/78 years old	USA	Diabetes mellitus type 2, peripheral vascular disease	Bloodstream infection (Haemodialysis associated)	Aminoglycosides, Ciprofloxacin	Aztreonam Ceftazidime, Cefepime, Piperacillin–Tazobactam,	Gentamicin, Ciprofloxacin	Complete Recovery
Alparslan et al. [133]	2013	M/12 years old	Turkey	End stage renal disease	Peritonitis (peritoneal dialysis infection)	N/A	N/A	Meropenem (initially 500 mg/L and then 200 mg/L), TMP-SMZ (TMP 320 mg/L-STX 1600 mg/L) PD catheter Removal	Complete Recovery
Chiu & Wang [134]	2013	M/34 years old	Singapore	None	Septic arthritis	Gentamicin, Meropenem, TMP-SMZ	Ceftazidime	TMP-SMZ (Oral)	Complete Recovery
Hagiya et al. [51]	2013	M/85 years old	Japan	Hepatocellular carcinoma, Liver cirrhosis	Bacteraemia	Amikacin, Colistin, Imipenem, Meropenem, Minocycline	Piperacillin, Piperacillin–Tazobactam, Aztreonam, Ceftazidime, Cefepime, Ciprofloxacin Levofloxacin, Gentamicin, TMP-SMZ	Cefcapene pivoxil (Oral)	Complete Recovery
Kumar et al. [135]	2013	M/45 days old	India	Neonate (congenital abnormalities)	Septicaemia and pneumonia	Ciprofloxacin, Gentamicin, Imipenem, Meropenem, Piperacillin–Tazobactam	Amikacin, Aztreonam	Meropenem	Complete Recovery
Mattos et al. [136]	2013	Multiple (12 Cases)	Brazil	Various	Endophthalmitis (Tubing following cataract surgery)	N/A	N/A	N/A	Complete Recovery
Mudshingkar et al. [137]	2013	M/Neonate	India	Neonate	Septicaemia	Amikacin, Imipenem, Meropenem	Ceftazidime, Cefepime, Gentamicin	Cefotaxime Gentamicin	Died
Mudshingkar et al. [137]	2013	M/Neonate	India	Neonate	Septicaemia	Amikacin, Imipenem, Meropenem	Ceftazidime, Cefepime, Gentamicin	Meropenem	Complete Recovery
Naik et al. [138]	2013	M/45 years old	USA	Hypotensive and Hypoxic	Pneumonia	Ciprofloxacin, Gentamicin Meropenem, Tobramycin	Ampicillin, Ampicillin–Sulbactam, Aztreonam, Cefazolin Cefepime, Cefotaxime, Ceftazidime, Piperacillin–Tazobactam, TMP-SMZ	Ciprofloxacin	Complete Recovery
Siti Rohani et al. [139]	2013	M/60 years old	Malaysia	Ischaemic heart disease, diabetes mellitus type 2, hypertension and end stage renal failur	Bacteraemia (catheter related)	Amikacin, Cefepime, Ciprofloxacin, Gentamicin Imipenem, Meropenem, TMP-SMZ	Ceftazidime, Piperacillin–Tazobactam Polymyxin-B	Imipenem 500 mg with Cilastatin (Intravenous 500 mg 12-hourly for 2 weeks) Catheters removal	Complete Recovery
Al-Naami et al. [140]	2014	M/15 years old	Australia	None	Retropharyngeal abscess	Amikacin, Cefepime, Ciprofloxacin, Gentamicin, Imipenem	N/A	N/A	Complete Recovery
Hernández-Torres et al. [141]	2014	M/73 years old	Spain	COPD, Hypertension, ischemic heart disease and chronic renal failure	Pneumonia	Ciprofloxacin, Doxicycline, Meropenem, Levofloxacin, TMP-SMZ, Tobramycin, Toimipenem	Amikacin, Aztreonam, Cephalosporins Piperacillin–Tazobactam	Levofloxacin (Oral)	Complete Recovery
Hernández-Torres et al. [141]	2014	M/38 years old	Spain	None	Bacteraemia (catheter related)	N/A	Amikacin, Aztreonam, Ciprofloxacin, Ceftazidime, Cefepime, Doxycycline Imipenem, Levofloxacin, Meropenem, Piperacillin–Tazobactam, TMP-SMZ, Tobramycin	Meropenem Teicoplanin	Complete Recovery
Hernández-Torres et al. [141]	2014	F/49 years old	Spain	Diabetes mellitus type 2 Adenocarcinoma	Biliary sepsis	Ciprofloxacin, Gentamicin, Imipenem, Levofloxacin, Meropenem	Amikacin, Cephalosporins, Piperacillin–Tazobactam, TMP-SMZ, Tobramycin	Piperacillin–Tazobactam Followed by Levofloxacin	Complete Recovery
Hernández-Torres et al. [141]	2014	M/61 years old	Spain	Liver cirrhosis	Transjugular intrahepatic portosystemic shunt device infection	Amikacin, Cefepime, Ciprofloxacin, Colistin, Gentamicin, Imipenem, Levofloxacin, Meropenem, Minocycline, Tigecycline, TMP-SMZ, Tobramycin	Ampicillin–Sulbactam, Aztreonam, Ceftazidime, Piperacillin, Piperacillin–Tazobactam	Meropenem	Died
Hernández-Torres et al. [141]	2014	F/56 years old	Spain	Acute myeloblastic leukaemia	Catheter-related infection	Ciprofloxacin, Imipenem, Levofloxacin, Meropenem, TMP-SMZ	Aminoglycosides, Aztreonam, Cephalosporins, Piperacillin–Tazobactam	Meropenem	Complete Recovery
Hernández-Torres et al. [141]	2014	5 Months old	Spain	None	Pseudo-bacteraemia	Amikacin, Carbapenems, Colistin, Doxycycline, TMP-SMZ	Aztreonam, Cephalosporins, Piperacillin–Tazobactam, Tobramycin	N/A	N/A
Khan et al. [142]	2014	F.53 years old	India	Chronic kidney disease, diabetes mellitus	Sepsis (catheter related)	Imipenem, TMP-SMZ	Aminoglycosides, β-lactams, Colistin, Quinolones	N/A	Died
Menezes et al. [143]	2014	F/Neonate	Brazil	Neonate with Cystic Fibrosis	Bacteraemia (catheter related)	Amikacin, Meropenem, TMP-SMZ	Ceftazidime	Amikacin and meropenem	Complete Recovery
Mrozek et al. [144]	2014	M/28 years old	France	Brain Trauma	Brain empyema	Carbapenems, Ciprofloxacin, Levofloxacin	Cefotaxime, Ceftazidime, Ticarcillin, Ticarcillin–Clavulanic acid, Piperacillin, Piperacillin–Tazobactam, Tobramycin, TMP-SMZ	Ciprofloxacin, Meropenem (IV for 6 weeks)	Complete Recovery
Quirino et al. [44]	2014	Multiple (19 Cases)	Italy	N/A	Bacteraemia	Amikacin, Ciprofloxacin, Gentamicin, Imipenem, Levofloxacin, TMP-SMZ	Ampicillin, Ampicillin–Sulbactam, Cefazolin, Cefepime, Cefoxitine, Ceftazidime, Ceftriaxone, Nitrofurantoin, Piperacillin–Tazobactam	N/A	N/A
Qasimyar et al. [145]	2014	M/Neonate	USA	Neonate	Sepsis (catheter related)	Amikacin, Levofloxacin, Meropenem	Β-lactams	Amikacin Meropenem (IV)	Complete Recovery
Wu et al. [146]	2014	M/35 years old	China	None	Neck abscess	Amikacin, Ciprofloxacin, Chloromycetin, Gentamicin, Meropenem, Imipenem, Levofloxacin Sulfamethoxazole, Tetracycline	Ampicillin, Ampicillin–Sulbactam, Amoxicillin–clavulanic acid, Aztreonam, Ceftazidime, Cefotaxime, Piperacillin Piperacillin–Tazobactam,	Levofloxacin	Complete Recovery
Cenkçi et al. [147]	2015	F/13 months old	Turkey	None	Bacteraemia, pneumonia	Cefepime, Gentamicin, Imipenem, Meropenem, Piperacillin–Tazobactam, TMP-SMZ	Cefotaxime, Ceftazidime, Ceftriaxone	Ceftriaxone	Complete Recovery
Hindilerden et al. [148]	2015	N/A	N/A	N/A	Bacteraemia	N/A	N/A	N/A	N/A
Patra et al. [149]	2015	M/54 years old	India	Guillain Barre Syndrome	Septicaemia	Amikacin, Ciprofloxacin, Gentamicin, Imipenem, Meropenem, Ofloxacin, TMP-SMZ, Piperacillin–Tazobactam	Ampicillin Aztreonam Ceftazidime, Ceftriaxone, Cefotaxime, Chloramphenicol, Piperacillin	Amikacin (15 mg/kg/day intravenous) Piperacillin–Tazobactam (3.375 g intravenous every 8 h)	Complete Recovery
Ashraf [150]	2016	F/58 years old	USA	Atrial fibrillation, End- stage renal disease with a failed kidney transplant, Coronary artery disease,	Septic shock, Infective endocarditis	N/A	N/A	Piperacillin–Tazobactum, vancomycin Followed by Meropenam	Complete Recovery
Haviari et al. [151]	2016	Multiple (3 Cases)	France	None	Bacteraemia (1 case)Urinary Tract Infection (2 cases)	Aminoglycosides, Carbapenems, Ciprofloxacin, Rifampin, Tigecycline, TMP-SMZ	Amoxicillin, Aztreonam, Cefalotine, Cefoxitine, Cefotaxime, Ceftazidime, Cefepime, Fosfomycin, Piperacillin, Ticarcillin	Ceftriaxone (1 g/day intravenously for 2/3 days) Ofloxacin (200 mg 2×/day orally for 10/21 days)	Complete Recovery
Jimenez and Antony, 2016 [152]	2016	M/40 years old	USA	Osteomyelitis and liver cirrhosis	Sepsis	Amikacin, Levofloxacin	Ampicillin–Sulbactam, Aztreonam, Cefepeme, Cefotaxime, Ceftazidime, Ceftriaxone	Levofloxacin	Complete Recovery
Kanjee et al. 2016 [153]	2016	F/60 years old	USA	None	Endophthalmitis	N/A	N/A	Moxifloxacin	Complete Recovery
Venkateswaran et al. [154]	2016	F/57 years old	USA	Herpetic keratitis and persistent central neurotrophic ulcer	Corneal ulcer keratitis (ocular detachment)	N/A	N/A	Tobramycin	Eye evisceration
Gigi et al. [155]	2017	M/18 years old	Israel	None	Osteomyelitis in the (Foot puncture)	N/A	N/A	Ciprofloxacin (Oral 750 mg 2/day) Clindamycin	Complete Recovery
Khasawneh & Yusef [156]	2017	F/Neonate	Jordan	Neonate	Sepsis (catheter related)	Amikacin, Imipenem, Meropenem, Piperacillin–Tazobactam	Ceftazidime, Cefipime, Gentamicin	Imipenem (25 mg/kg twice daily) Amikacin (15 mg/kg)	Complete Recovery
Rastogi & Mathur [23]	2017	M/58 years old	India	Severe head injury	Septicaemia with meningitis (catheter related)	Amikacin, Cefepime–Tazobactam, Colistin, Tigecycline TMP-SMZ	Ceftazidime, Cefepime, Cefoperazone–Sulbactam, Chloramphenicol, Ciprofloxacin, Imipenem, Meropenem Piperacillin–Tazobactam,	Cefepime–Tazobactam (1.12 gm) Amikacin (400 mg) (injection every 12 h)	Complete Recovery
Torres Aguilera et al. [157]	2017	F/88 years old	Spain	Diabetic and hypertensive, with significant vascular disease	Bacteraemia (catheter related)	N/A	N/A	Ciprofloxacin Catheter removal	Complete Recovery
Torres Aguilera et al. [157]	2017	M/84 years old HA	Spain	Diabetic nephropathy	Bacteraemia (catheter related)	N/A	N/A	Initial treatment Ciprofloxacin Followed by Meropenem and TMP-SMZ Catheter removal	Complete Recovery
Cipolla et al. [158]	2018	Multiple (20 Cases)	Argentina	N/A	Bacteraemia	N/A	N/A	N/A	N/A
Hafeez et al. [159]	2018	M/64 years old	USA	Alcohol abuse, Hypertension	Pneumonia	N/A	N/A	Ciprofloxacin followed by Meropenem	Complete Recovery
Montaña et al. [61]	2018	Multiple (6 Cases)	Argentina	N/A	Pseudo-bacteraemia	N/A	Meropenem	N/A	N/A
Zhu et al. [160]	2018	Multiple (11 Cases) HA +CA	China	Various	Bloodstream infection (catheter related)	Ciprofloxacin, Levofloxacin, Imipenem, TMP-SMZ	Ampicillin, Cefoperazone–Sulbactam, Ceftazidime, Cefuroxime, Cefazolin, Piperacillin	Various	Complete Recovery (10 Cases) Died (1 case)
Caroleo et al. [161]	2019	Multiple (4 Cases) HA	Italy	Cancer	Catheter-related bloodstream infections	N/A	N/A	N/A	N/A
Grabowska-Markowska et al. [162]	2019	M/13 years old	Poland	Neurodegenerative disorder	Bacteraemia	Imipenem, Meropenem	Ceftazidime, Piperacillin–Tazobactam	None	Complete Recovery
Kang et al. [163]	2019	F/53 years old	Korea	None	Keratitis	Ciprofloxacin, Gentamicin	Amoxicillin, Ampicillin, Benzylpenicillin, Cefepime, Ceftazidime, Ceftriaxone, Imipenem, Piperacillin	Gentamicin	Complete Recovery
Roussotte et al. [164]	2019	F/53 years old HA	France	Facial oedema	Catheter-related infection associated with superior vena cava	Amikacin, Ciprofloxacin, Ertapenem Gentamicin Imipenem, Meropenem, Moxifloxacin, TMP-SMZ, Tobramycin	Ampicillin, Amoxicillin, Amoxicillin–Clavulanate, Aztreonam, Cefepime, Cefoxitine, Cefotaxime, Ceftazidime, Fosfomycin, Nalidixic acid, Piperacillin, Piperacillin–Tazobactam Ticarcillin–Clavulanate	Imipenem–Cilastine, Ciprofloxacin Catheter removal	Complete Recovery
Arimuthu and Seong Lim [165]	2020	M/24 years old HA	Malaysia	Dengue viral fever	Bacteraemia (catheter related)	Ciprofloxacin, Gentamicin, Imipenem Meropenem, Tigecycline	Ceftazidime, Cefepime, Piperacillin–Tazobactam, Polymyxin B, TMP-SMZ	Meropenem (2 g thrice a day) After failure Catheter removal Ciprofloxacin (Intravenously 400 mg thrice a day)	Complete Recovery
Arimuthu and Seong Lim [165]	2020	M/64 years old HA	Malaysia	Diabetes, end stage renal disease, Hypertension, Ischemic dilated cardiomyopathy	Bacteraemia (catheter related)	Ciprofloxacin, TMP-SMZ	N/A	Ciprofloxacin	Complete Recovery
Bratschi et al. [166]	2020	M/70 years old CA	Switzerland	None	Hand infection	N/A	N/A	Surgical debridement Amoxicillin–clavulanic acid (empirically) Cefepime (2 g 3 times/day intravenously for 15 days) Co-trimoxazole (960 mg 3 times/day orally for 2 weeks)	Complete Recovery
Ko et al. [167]	2016–2020	Multiple (5 cases)	Korea	Various (Pneumonia, Hypertension, Diabetes mellitus	Various	Ciprofloxacin, Levofloxacin, TMP-SMZ	Aztreonam, Cefepime, Cefotaxime; Ceftazidime, Piperacillin, Piperacillin–Tazobactam Ticacillin–Clavulanic acid		Complete Recovery in 3 patients Death in 2 patients

M, Male; F, Female; N/A, Not Available; CA, Community Acquired; HA, Hospital Acquired; TMP-SMZ, Trimethoprim–sulfamethoxazole. * Antibiotic susceptibility testing was carried out using a variety of methods including disk diffusion testing, agar and broth dilution testing and E-testing methods.

**Table 5:** Incidences of *Ochrobactrum* spp. (excluding *Ochrobactrum anthropi*) infection from 1998–2020. Main characteristics of the case reports.

Author (Ref) Bacteria	Year	Sex/Age	Country	Co-Morbidity	Type of Infection	Susceptible to *	Resistance to *	Treatment	Outcome
Möller et al. [8] *Ochrobactrum intermedium*	1999	F/45 years old	The Netherlands	Liver transplant patient	Bacteraemia	Ciprofloxacin, Imipenem, TMP-SMZ	Amoxicillin, Cefuroxime, Cefotaxime, Ceftazidime, Colistin, Piperacillin, Polymyxin B, Tobramycin	Imipenem Tobramycin	Complete Recovery
Apisarnthanarak et al. [171] *Ochrobactrum intermedium*	2005	M/74 years old	Thailand	Bladder cancer	Bacteraemia	Aminoglycosides, Carbapenems, Fluoroquinolones, TMP-SMZ	N/A	Ciprofloxacin Imipenem	Complete Recovery
Vaidya et al. [106] *Ochrobactrum intermedium*	2006	M/49 years old	USA	None	Pelvic abscess	Gentamicin, Imipenem, Levofloxacin, TMP-SMZ	Cefepime, Tobramycin	Levofloxacin, Metronidazole	Complete Recovery
Teyssier et al. [36] *Ochrobactrum pseudintermedium*	2007	Multiple (2 cases)	France	N/A	ICU patient	Ciprofloxacin, Gentamicin, Nalidixic acid, Ofloxacin, Pefloxacin, Rifampicin	Fosfomycin	N/A	N/A
Dharne et al. [172] *Ochrobactrum intermedium*	2008	M/	India	N/A	Stomach isolate from non-ulcer dyspeptic patient	N/A	N/A	N/A	N/A
Jacobs et al. [173] *Ochrobactrum intermedium*	2013	M/34 years old	USA	None	Endophthalmitis (metallic intraocular foreign body contamination)	Ciprofloxacin, Levofloxacin, TMP-SMZ	Amikacin, Ampicillin, Ampicillin–Sulbactam, Ceftazidime, Ceftriaxone, Gentamicin, Piperacillin–Tazobactam, Tobramycin	Moxifloxacin	Complete Recovery
Hirai et al. 2016 [59] *Ochrobactrum intermedium*	2016	M/86 years old	Japan	N/A	Pneumonia (catheter related)	Amikacin, Ciprofloxacin, Imipenem, Levofloxacin, Meropenem, Minocycline	Aztreonam, Ceftazidime	Ampicillin–Sulbactam followed by Meropenem (2 g/day)	Complete Recovery
Borges et al. [174] *Ochrobactrum oryzae*	2016	M/86 years old	Brazil	Hypertension, type II diabetes mellitus, dyslipideamia, end stage renal disease	Bloodstream infection	Amikacin, Ciprofloxacin, Imipenem, Meropenem,	Polymyxin B	Imipenem	Complete Recovery
Hong et al. [175] *Ochrobactrum tritici*	2016	M/70 years old	Korea	Cholangiocellular carcinoma	Bacteraemia, Cholecystitis	N/A	Ceftriaxone, Cefepime, Ticarcillin	Cefoperazone–Sulbactam (2000 mg every 12 h), Metronidazole (500 mg every 8 h)	Complete Recovery
Bharucha et al. [176] *Ochrobactrum intermedium*	2019	M/23 years old	UK	Undergoing haemodialysis	Endocarditis (catheter related)	Ertapenem, Meropenem, Tigecycline	Ciprofloxacin, Colistin, Fosfomycin	Meropenem (1 g iv twice daily), Minocycline (100 mg iv twice daily)	Complete Recovery
Cho et al. [177] *Ochrobactrum pseudogrignonense*	2020	M/44 years old	Korea	Hypertension, diabetes mellitus, dilated cardiomyopathy	Bacteraemia	Amikacin, Ampicillin–Sulbactam, Ceftazidime, Cefepime, Cefotaxime, Ciprofloxacin, Colistin, Gentamicin, Imipenem, Meropenem, Minocycline, TMP-SMZ	Aztreonam, Piperacillin, Piperacillin–Tazobactam	Vancomycin and Piperacillin–Tazobactam Followed by Meropenem	Complete Recovery

M, Male; F, Female; N/A, Not Available; CA, Community Acquired; HA, Hospital Acquired; TMP-SMZ, Trimethoprim–sulfamethoxazole. * Antibiotic susceptibility testing was carried out using a variety of methods including disk diffusion testing, agar and broth dilution testing and E-testing methods.
